# Building and analyzing an innovative community-centered dengue-ecosystem management intervention in Yogyakarta, Indonesia

**DOI:** 10.1179/2047773212Y.0000000062

**Published:** 2012-12

**Authors:** Susilowati Tana, SittiRahmah Umniyati, Max Petzold, Axel Kroeger, Johannes Sommerfeld

**Affiliations:** 1 Center for Health Policy and Social Change, Sleman, Daerah Istimewa Yogyakarta, Indonesia*; 2 Department of Parasitology, Faculty of Medicine, Gadjahmada University, Indonesia; 3 Sahlgrenska Academy at University of Gothenburg, Sweden; 4 Special Programme for Research and Training in Tropical Diseases (TDR), World Health Organization, Geneva, Switzerland; 5 Liverpool School of Tropical Medicine, Liverpool, UK

**Keywords:** Dengue, community participation, empowerment, waste management

## Abstract

**Background and Objectives:**

Dengue is an important public health problem in Yogyakarta city, Indonesia. The aim of this study was to build an innovative community-centered dengue-ecosystem management intervention in the city and to assess the process and results.

**Methods:**

For describing the baseline situation, entomological surveys and household surveys were carried out in six randomly selected neighborhoods in Yogyakarta city, documents were analyzed and different stakeholders involved in dengue control and environmental management were interviewed. Then a community-centered dengue-ecosystem management intervention was built up in two of the neighborhoods (Demangan and Giwangan) whereas two neighborhoods served as controls with no intervention (Tahunan and Bener). Six months after the intervention follow up surveys (household interviews and entomological) were conducted as well as focus group discussions and key informant interviews.

**FIindings:**

The intervention results included: better community knowledge, attitude and practices in dengue prevention; increased household and community participation; improved partnership including a variety of stakeholders with prospects for sustainability; vector control efforts refocused on environmental and health issues; increased community ownership of dengue vector management including broader community development activities such as solid waste management and recycling.

**Conclusion:**

The community-centred approach needs a lot of effort at the beginning but has better prospects for sustainability than the vertical “top-down” approach.

## Introduction

Dengue infection is a significant and increasing public health problem in Indonesia and neighboring countries. The disease is endemic in Yogyakarta city, with all 45 *kelurahan* reporting cases (*kelurahan* is a level of formal governance in urban areas of Indonesia, equal to a village in rural areas) and reported an annual average rate of 16.8 dengue cases per 10,000 inhabitants from 2005 to 2007 (Yogyakarta City Health Office, 2008). Reduction of mosquito breeding in houses and public spaces through various measures including larval growth inhibitor and interventions against adult mosquitoes requires a continuous effort by the community (Parks *et al*., 2004, Nathan *et al*., 1991). Reports from various countries indicate that vertical *Ae. aegypti* control activities are not sustainable (Nathan & Knudsen, 1991, Winch *et al*., 1992, Rosenbaum *et al.*,1995) and that isolated vector control initiatives do not promote behavioral change (Castle *et al*., 1999, Parks *et a*l., 2004, Kay & Vu, 2005), suggesting that sustained active community participation in source reduction and environmental management are probably the most effective at preventing dengue epidemics. The findings of Toledo *et al*. (2008) corroborated the importance of behavioral change and the need to actively involve the community in the design and implementation of strategies to control *Ae. aegypti*.

Bermejo and Bekui (1993) and Rosenbaum *et al*. (1995) showed that purely technical interventions limit the perceived self-reliance of the community and reinforce the belief that the government is responsible for vector control but Heintze *et al*. (2007) showed in a systematic literature review that the evidence base for community involvement in dengue vector control was still weak at that time. However, a new study by Toledo *et al*. (2008) revealed that communities themselves must be involved in environmental risk reduction. Top–down deployment of technical tools without active involvement of the community has a temporary effect and does not lead to the behavioral changes necessary for sustainable *Ae. aegypti* control. Nam *et al*. (2005) installed successfully in Vietnam through community action groups a programme of biological dengue vector control using mesocyclops. Building on these experiences and observations, this study was aimed at building and analyzing an innovative community-centered dengue-ecosystem management intervention in Yogyakarta city in order to identify a more sustainable strategy for dengue vector management.

## Methods and study sites

### Conceptual Framework

The assumption of this study was that community participation in dengue vector control, particularly when using methods of environmental management, will increase the likelihood of program sustainability. Bottom-up deployment through active involvement of the community will have a lasting effect if the existing structures are strengthened and supported (in the case of our study area community leaders, community volunteers and community assemblies called community forums) then the community will be enabled to decide by themselves the type of intervention they prefer and the role they assign to facilitators (in our case the research team). The likelihood of programme sustainability increases with increased community involvement in terms of improved knowledge about what is the problem and what can be done, and if new practices enter into their daily or weekly routine. In our study five indicators for assessing community participation in health programs (Draper *et al*. 2010) were applied in a descriptive way: leadership of the community and of other stakeholders when introducing the intervention; planning and managing partnerships between the community and professionals; women’s involvement; gaining financial independence and “grip” on further intervention design; monitoring and evaluation by the community; examining how intended beneficiaries are involved in programmed activities. The effect of a participatory intervention package was assessed using as outcome variables: financial independence, increased community participation, better knowledge and improved practices (see box below). The impact on vector densities was to be roughly assessed through entomological surveys but no attempt for assessing the significance of vector reduction was made due to the low number of study clusters and external factors.

**Table:** 

Dimension	Indicators for expected outcomes
**Resources**	Resource mobilization by the community
**Participation**	Leadership within the community
	Community involvement in decision making
	Financial independence
**Knowledge**	Dengue prevention measures are known
**Attitude**	Peoples’ perceived roles and responsibility of government and community on dengue prevention
**Practice**	Dengue prevention measures implemented
**Entomology indices**	Pupae per person index
**Sustainability**	Lasting program without project support

### Geographic location of the study area, climate and dengue season

Yogyakarta city has a tropical marine monsoon climate. It lies just above sea level at 07°49’26′–07°15’24′ south latitude and 110°24’19′–110°28’53′ east longitude. The highest humidity level in 2007 occurred in April (87% relative humidity) and the lowest in September (73%). The average annual temperature was 26.7°C. The average monthly rainfall for the year 2008 was 80.18 mm, with a range of 210.80 mm in February and 0mm in August (0 mm). (Department of Transportation Yogyakarta Special Province, 2009). During the wet seasons (November to February) increased numbers of dengue are being reported (“dengue season”).

### Socio-demographic conditions and dengue

The city of Yogyakarta on the island of Java is the capital of smallest province of Indonesia (excluding Jakarta). The 2005 population survey indicated the total population of Yogyakarta city to be 435, 236 with an average population density of 13,392 people per square km (Yogyakarta City Central Statistics Board 2005), this level of crowding being typical for the large Indonesian cities. Our previous study in 12 randomly selected neighborhoods showed that the housing conditions are generally satisfactory and that the large majority of people belong to low- and middle-class neighborhoods (Arunachalam *et al*. 2010) Main occupations in the city are employees, small scale traders and private enterpreneurs. The minimum wage is 60 USD. Each year the city hosts several trade shows, exhibitions and trade fairs, thereby attracting millions of visitors and exhibitors throughout the year, posing the threat of introducing dengue viruses into the local population. This may partly explain why Yogyakarta had for the last three decades one of the highest dengue hospitalization rates in Indonesia and a considerable number of dengue outbreaks. Other factors contributing to the elevated dengue transmission risk are the water storage pattern by the population and the discarded tyres in the environment–typical for most urban areas in Indonesia (see study by Umniyati *et al*., 2006).

### Public infrastructure

Yogyakarta city government provides piped water to roughly one third of neighborhoods but many households still rely on ground water from wells and pumps because they have to pay for the drinking water (Arunachalam *et al*. 2010). Garbage and waste is collected once per week in almost all parts of the city. Some neighborhoods, particularly those living along river banks, still throw their garbage into the rivers.

### Vector control and communication strategies in Yogyakarta

The dengue control programme in Yogyakarta city is largely the responsibility of the district health office. Dengue prevention and control have become a priority consisting of disease surveillance, vector control and community education. Vector control is almost exclusively implemented by larvae control workers who are hired within the communities by the district health officer for certain periods of time; they are usually young and have little training in larvae control and are far too few to cover the whole city at short intervals. The community education programme is limited in terms of quantity and coverage. The 3M campaign (Menguras, Menutup, Mengubur – clean, close and bury water containers) is a nationwide clean up campaign which is well known both in the country and internationally but has not been evaluated formally.

### Sampling of study sites within Yogykarta

In the baseline study 12 neighborhoods (“study clusters”) with about 100 households each were randomly selected in Yogyakarta city using grid sampling (see sampling strategy in Focks 2004 and Arunachalam *et al*. 2010) For the intervention study, 3 areas (which exceeded the original study clusters in size as they respected socio-political boundaries) with a high incidence rate of reported dengue (Demangan, Tahunan, and Giwangan) and 3 areas with a lower incidence rate of reported dengue cases (Baciro, Suryatmajan, and Bener) were randomly selected from the 12 original sites using the lottery method, attempting a fairly representative sample of the city.

### The intervention package

The intervention package to build up community-centred dengue eco-system management in Yogyakarta was applied in the intervention neighborhoods.

The intervention package included:

1. Community involvement and empowerment through meetings, forums, community leaders (cadres), neighborhood assemblies, women associations, primary schools. Community active participation was facilitated through community forum. Community forum is forum at neighborhood level. There are two community forums at the study sites i.e Demangan and Giwangan. They were led by formal/informal community leaders. The members were the local community.
2. Involvement of other partners: environmental health forum, local political authorities, health facilities and institutions, public services (water and sewage), NGOs (particularly Tahija Foundation).
3. Production of intervention tools such as communication materials and development of awareness campaigns in schools.

As this was a dynamic interactive process whereby findings were immediately discussed with stakeholders and appropriate actions were defined, a more detailed description will be presented in the results section.

### Data collection and time line

The here described intervention study was designed to analyse the process and results of a novel intervention package. The data collection was done by four members of the research team, the entomological surveys were conducted by selected and re-trained larval workers under the supervision of the research team. The data collection included the following: (1) Collection of institutional data regarding reported dengue cases and vector control strategy; (2) Entomological surveys using standard operational procedures for larval surveys and pupal/demographic surveys in six neighborhoods (clusters in 2007); (3) Formal household interview surveys in six neighborhoods and 423 houses; (4) The household surveys and entomological surveys were repeated in 2008 in 401 houses (21 drop outs) in order to get information on the changes achieved by the intervention; (5) 15 Focus Group Discussions (FGDs) were held in all study clusters with community members during the baseline assessment, following the method suggested by Khan and Manderson (1992); (6) In-depth interviews with key informants and stakeholders.

Household interviews, FGDs and in-depth interviews were focusing on people’s awareness of dengue being a public health threat, role of communities, government and other stakeholders in dengue prevention as well as willingness to participate in preventive activities.

The first entomology survey was carried out in in the dry season (September 2007) in 1,047 houses of the original 12 clusters from 6 *kelurahans*, then it was repeated in the wet season in January 2008, in 1,044 houses.

The baseline assessment was done from October to November 2007, the intervention including continuous assessment of progress was done from June 2008 to May 2009. The outcome analysis was done from October to December 2009.

### Data analysis

A categorical analysis was applied to documents, key informant interviews and focus group discussions, and findings were presented in a descriptive way. Quantitative survey data were mainly analysed by comparing the findings before and after applying the intervention package and by comparing control and intervention clusters.

Entomological indicators were the following: House index (HI): percentage of houses infested with larvae and/or pupae; Container index (CI): percentage of water-holding containers infested with larvae or pupae; Breteau index (BI): number of positive containers per 100 houses; Pupal index: number of pupae per 100 houses (WHO, 1999); Productive container types “were those containers which produced together more than 70% of all pupae encountered. Nathan *et al*. (2006) suggested pupal/demographic surveys to inform dengue vector control about productive container types for targeted interventions. Pupae counts, determined by pupal-demographic surveys (Focks and Alexander 2006), were used as a proxy for adult vector counts as around 80% of pupae develop to adult mosquitoes (Focks 2004). In contrast, larval indices (HI, CI, BI) indicate the presence or absence of dengue vectors but are not a measure for vector density (Nathan *et al*. 2006).

While no statistical significance testing could be done due to the low number of clusters, the difference from baseline to follow up was calculated as “% at follow up - % at baseline” for the mean values of clusters and denoted DiffInt and DiffCont for the intervention and control areas, respectively. The “difference-of-differences” was calculated as DiffInt – DiffCont. A positive difference-of-differences shows that the intervention had an effect on vector densities, but due to the study design it was not possible to assess if the difference was statistically significant.

### Ethical aspects

Participants in the interviews and FGDs signed a written consent form before answering questions. The study was approved by the Ethical Review Committee of WHO and the IRB in Yogyakarta.

## Results

In the following we will first outline the baseline assessment (situational analysis including entomological study and qualitative study with community members). Secondly we will describe the development of the intervention package and its different elements, which was based on the community study and stakeholder analysis (as part of the situational analysis) as well as on the entomological assessment. Thirdly we will describe the results of the intervention regarding community participation in control efforts, improving knowledge and preventive practices and finally the potential impact on vector densities, although the study design did not allow for measuring the significance levels of reduced vector abundance.

### 1. Situational analysis in the pre-intervention phase

#### Determining productive container types through pupal-demographic surveys

The entomology survey in the dry season in September 2007 showed that the most productive containers for dengue vectors were the cement/steel tanks which produced 88.6% of all pupae (Table 1). In the wet season in January 2008, the most productive containers were again the cement/steel tanks (53.1% of all pupae) with the addition of drums (17.1% of pupae), flower vases (15.5%) and buckets (11.6%) indicating at which container types the proposed intervention strategy had to focus. The pupal and larval indices, and incidence rate of reported dengue cases is presented in Table 2 showing: a) considerably higher indices in the wet season, b) good correlation among pupal indices (PI and PPP) and much less among larval indices; c) a good correlation between pupal indices and reported incidence.

**Table 1:** Total number of pupae by type of container in the dry season in 2007 and in the wet season in 2008, in six neighborhoods (clusters)

		Dry season		Wet season	
	Type of	No of	%	No of	%
No	Container	pupae	pupae	pupae	Pupae
1	Cement/Steel tank	1223	88.6	995	53.1
2	Bowl	48	3.5	45	2.4
3	Bucket	47	3.4	218	11.6
4	Flower vase	32	2.3	291	15.5
5	Water tower	17	1.2	0	0
6	Bird drinking water container	10	0.7	3	0.2
7	Chicken drinking water container	4	0.3	0	0
8	drum/barrel	0	0	0	17.1
	Total	1381	100	1873	100

**Table 2:** Entomology indices (House Index (HI), container index (CI), Breteau Index (BI), Pupa Index (PI), pupae/person(PPP), and Incidence Rate (IR) in the dry season in September 2007 and in the wet season in 2008, in six neighborhoods (clusters)

	HI (%)		CI (%)		BI		PI		Pupae/ person		IR
Cluster	Dry	Wet	Dry	Wet	Dry	Wet	Dry	Wet	Dry	Wet	Dry
Demangan	21.20	25.90	5.30	7.70	39.3	42.0	429	180	0.82	0.35	12.2
Tahunan	30.70	33.60	7.80	10.40	37.1	58.5	168	158	0.35	0.33	17.2
Giwangan	18.50	37.60	4.50	11.20	24.9	63.4	108	357	0.26	0.87	19.3
Baciro	18.20	44.50	5.80	15.40	21	76.3	47	272	0.10	0.56	7.3
Suryatmaja	17.20	13.70	8.80	7.60	24.7	29.0	9	181	0.01	0.47	1.5
Bener	12.90	27.70	5.80	9.40	15.3	47.5	17	139	0.04	0.31	0.9
Average	19.78	33.10	6.33	10.28	27.05	55.3	155	223	0.26	0.48	9.7

Presentation of entomological indices in section “data analysis”. IR = incidence rate of reported dengue per 100,000 population.

#### Lack of community involvement and knowledge at baseline

In-depth interviews with city government officers showed the following: Although there are defined community structures with leaders, volunteers and assemblies (forums) the 3M campaign (see introduction) is often not implemented by community members and is therefore limited in scope. The community sees dengue control as a government responsibility, and has limited knowledge about mosquito breeding places. The most common community education programme is to show banners and distribute leaflets at community health centres and district health offices but such one-way communication is not very effective and is limited in coverage. Other promotion activities include: “My house is clean” stickers, posters and leaflets on mosquito source reduction, leaflets on dengue case management, press releases, radio and TV spots, direct community education and training of cadres in each *kelurahan* twice a year. These promotion efforts have resulted in increased community knowledge about dengue vector control but not in improved behavior concerning source reduction.

Focus group discussions and in-depth interviews with community leaders of all study clusters showed that people were concerned about dengue which had affected most families in the study area. The discussions at the beginning highlighted also the widespread misconceptions regarding the role of the government in dengue vector management (“this is their responsibility”) and lack of understanding of peoples’ own contribution; this meant that in spite of a reasonable understanding of dengue transmission and the disease (see later Table 4) there was no strong feeling that their own contribution would be important. Additionally the Tahija Foundation project (a charitable enterprise by the Tahija family) had probably strengthened this concept of “others being responsible for vector control”: The Tahija project distributed pyriproxifen through trained field workers who were hired to visit and check homes on a massive basis; there were also “vertical” educational activities through mass media (radio, newspaper, banners, etc.), information for community health centres and local village authorities, and information for householders regarding the application of pyriproxifen. The understanding of peoples’ “paternalistic” way of thinking helped design the intervention strategy (Summary in Table 3).

**Table 3:** Social factors contributing to the low participation seen at baseline

Reasons for low community involvement at baseline	Strategies and approaches proposed for the intervention study
Misconceptions regarding government role in dengue prevention and vector control	Raising awareness through community education programmes and household visits, community cadres and media.
Lack of comprehensive understanding about dengue prevention and ecosystem management	Enhancing community leadership for dengue prevention and ecosystem management.
No real community action in spite of reasonable dengue knowledge	Strengthening community organization and leadership for dengue prevention so the community became more active, through systematic development of a community mobilization and facilitation process.

**Table 4:** Increased community participation after the intervention according to a set of indicators (as discussed and agreed upon in stakeholder meetings)

Indicators of participation (according to Draper *et al*. 2010)	Increased community participation after implementing the intervention package
Leadership Community introducing the intervention	Programme led by community members who were selected by the community. Researchers gave technical assistance or training if necessary at the beginning.
Planning and management taken over by the community	Researchers had a facilitator role at the beginning. Later on, community members took the lead, defining priorities and managing the programme. Local people learned skills for monitoring and evaluation.
Women’s involvement	Women actively participated particularly through women groups. Women and men together made the decisions.
Programme development to achieve financial independence and mastering the programme design	Community members worked to mobilize local resources in order to replace external funding by their own resources. Community members designed the programme with technical advice as needed. Decision making involved women.
Monitoring and evaluation by the community	The community carried out participatory evaluation using agreed local indicators. The community was actively involved in monitoring and deciding how to respond to the findings.

### 2. Intervention package

#### Identifying partners and building consensus on the proposed intervention

The stakeholder analysis had identified a number of potential actors in a community-centred dengue strategy. These included: (1) Community-based environmental health forums (*forum lingkungan)* of interested individuals who attend neighborhood assemblies at *rukun warga* (RW) (community) level; (2) Community leader (cadres) who were elected by the communities; (3) Voluntary community workers interested in environmental issues and health; (4) Women’s associations (*pendidikan kesejahteraan keluarga*); (5) Local political authorities at different governance levels (*rukun tetangga* - a subdivision of the *kelurahan* and composed of about 50 families each, RW, village, sub-district, city); (6) health sector institutions (district health centres, community health centres; larvae workers); (7) city office for environmental affairs (Department of the environment) dealing with public services such as water and sewage; (8) public utility department; (9) non-governmental organizations (e.g. Tahija Foundation); (10) primary schools.

Through meetings with and among partners and through personal visits consensus was built regarding the shape of the future intervention package. The results were then presented to local authorities to obtain their support.

#### Building community empowerment and costs

A community mobilization and facilitation process, through neighborhood assemblies, was used to build up partnership and a climate of “working together”. In the first phase of developing and implementing the intervention package, the following activities were a priority:

1. Assessing community capacity for collaboration and co-financing; through periodic meetings with community organizations.
2. Capacity building and mentoring of community leaders: community members who were interested in health and environmental issues, elected by the community, and willing to work voluntarily for their neighborhood were trained in facilitation and communication skills and how to work and communicate with the community as a whole and with particular community groups (women’s and self-help organizations, schoolchildren). They learned to inform the community about dengue prevention, to advocate for local ecosystem initiatives, and to liaise with the authorities.
3. Strengthening community forums. Two community forums were reshaped in the intervention sites, one of them within the legal framework. Each forum set up periodic meetings to participate in identifying problems, programme development, and in monitoring and evaluation. During the community intervention work, the research team provided logistics support for specific meetings as well as limited financial support (see below) but the community forum was free to decide how it should be allocated. This support was particularly for meeting or community work and not salary. This limitation of support aimed at preventing subsidies or dependency to the external parties, which reduce sustainability. Later on, the responsibilities were taken up by the forums themselves: After the support finished, each forum had some savings and continued to mobilize resources through community membership fees and/or selling recycled community products. Additionally, the Department of the Environment provided two half-day training sessions for community forums on household solid waste management (sorting and recycling) and making compost. This program was decided by community forum. Although there is no evidence yet that waste contributes to productive breeding sites, it is part of the vision of community forum, which does not want to only focus on dengue. After the training, the community boards (community members who volunteered to lead the initiative) and community leaders contributed to monitoring, follow up and evaluation of the programme, and reported on the results and discussed any problems in their community meetings. An additional training unit on how to make use of recycled plastic bags was developed. Each community forum received 4–5 training sessions, each lasting 1–2 hours and held every two weeks. Later on, the more skillful participants became trainers for the rest of group.
4. Training and capacity building: Training was given in a very informal way and encouraged interactive learning. The venue was a public hall or house of a community member, discussing mosquito control measures with emphasis on non-insecticidal interventions such as larvivorous fish, well covers, waste removal and recycling activities. Training was just the starting point for capacity building because, after the initial training, it was important for each community forum to conduct periodic follow-up training sessions and ensure that tools and skills were being used to achieve improved health and environment.
5. Community ownership: This was strengthened by a series of thematic community self-help programmes, particularly targeting vector breeding places in productive containers and in public spaces (as identified at baseline). The range of activities was decided by the forum and did not always focus exclusively on dengue, but could be related to the environment and economic issues. Every month community members worked together for six hours under the leadership of one member of the community to improve the environment of their neighborhood.
6. School health and outreach activities: Information was introduced into the routine school programmes through headmasters and teachers. The school programme was linked with existing programmes within and beyond the school; it was integrated into the existing school health programme under the local community health centre. Trained schoolchildren were encouraged to educate their peers and families. Community health centres were also involved, allowing the intervention package to be integrated into the existing health programme.

Financial contributions in the first six months of the project to each community were the following: a) USD 20–30 (in exceptional cases up to 50 USD) per month to support forum meetings and some expenditures for communication materials and mobilization; b) Minimum wage of 60 USD for supporting community volunteers. After this initial period communities identified alternative sources of financial support.

#### Designing locally relevant and culturally acceptable communication materials

As part of the capacity building process, dengue prevention materials were provided for community leaders and facilitators, schoolchildren and householders. A short informative video on breeding places and larvae/pupae elimination strategy was developed together with community members and used during community education sessions. A poster showing how humans cause environmental problems and interact with mosquitoes was developed jointly with community forums and health authorities and was displayed in public areas of the intervention clusters; likewise a pocket book on dengue prevention for community cadres and householders was jointly developed and disseminated. As part of the school programme, children were facilitated to create their own media and wall magazines about dengue prevention.

#### Delivering the intervention during the different project phases

In the early phase of project, local stakeholders were receivers of information and implementers but in the later stages they were in the driving seat and the research team acted as facilitator or observer. After having achieved the collaboration of a number of partners (stakeholders) and created the sense of “working together” the partners took over including: continued strengthening of local community organizations; facilitating local leadership; community education programmes through community leaders and forums as well as the media; informing households through home visits of larvae workers; encouraging spontaneous self-help programmes to become more organized; linking inter-sectoral collaborations to community and school initiatives; training and facilitating schoolchildren to become ambassadors and leaders for their peers in the dengue initiative; facilitating community forums to formalize their organizations; communicating with local authorities, particularly for sharing resources.

The forums also gradually expanded into areas beyond the intervention sites, following the networks of community interaction. Besides the community leaders, some members of the community forum board were chosen to deal with dengue prevention in their official role in the neighborhood, e.g. as members of legislative bodies or a local authority. Occasional visits to the intervention communities during 12 months after finishing the project showed that they were still actively engaged in dengue prevention and other health matters. Visits 24 months after finishing the project in one community (Giwangan) showed that people were still working on dengue prevention and improving health.

### Results of the community-centred intervention

#### Results on community participation

Table 4 shows that six months after the start of the intervention phase the entire program (planning, implementation and evaluation) was led by the community with the involvement of women groups. In order to be sure that the findings reflect the perceptions and experiences of all stakeholders involved in the programme, the findings were discussed and agreed upon in community meetings, FGDs with stakeholders and informal in-depth interviews with local politicians.

#### Results on people’s knowledge

The household surveys in the study neighborhoods showed that after the intervention, respondents in intervention neighborhoods were more knowledgeable about dengue and dengue prevention than respondents in the control group: respondents expressing the need for water container management and other vector control measures increased substantially (Table 5).

**Table 5:** Knowledge about dengue prevention (“How can you prevent dengue?” without specifying the options) before and after the intervention

Suggested actions	Before		After		Difference of difference
	Control N = 210	Intervention N = 213	Control N = 200	Intervention N = 201	
Do water container management at home	31.90%	35.01%	27.75%	71.40%	40.54%
Put larvivorous fish into water tanks	4.50%	7.20%	5%	51.70%	44.00%
Put mosquito wire screens on windows and doors	0.50%	2.70%	0.50%	45.80%	43.10%
Put pyriproxifen into the water	26%	28.30%	5.50%	41.30%	33.50%
Spray insecticide in your home	7.50%	6.70%	3%	10.40%	8.20%

In an open-ended question respondents explained that “water container management” included: destroying trash containers, storing trash water containers, burying trash water containers, selling trash water containers, covering water containers with lids, rubbing the inside walls of water containers, and turning the containers upside down. The need for other interventions e.g. using abate sand, using pyriproxifen in water containers, destroying adult mosquitoes by electrical devices, and putting mosquito wire screens on windows and doors, was also expressed more frequently. In the question about “other control activities” respondents at baseline had mentioned only “mosquito coils”, “eat fruits” and “physical exercise”, while respondents in the intervention neighborhoods mentioned in the follow-up survey: “cleaning water containers”, “sort solid waste”, “clean stagnant water”, “maintain good environmental conditions”.

#### Results on people’s practices

The intervention increased the percentage of families who participated in different community actions for dengue prevention (Table 6). Most frequently, cleaning up the environment, participating in meetings to discuss dengue, and checking water containers in houses and public spaces were mentioned. The percentage of families who were protecting or destroying breeding places increased in the intervention group (difference of differences  =  6.3%) but this proportion was already high at baseline because of the pyriproxifen campaign conducted by the Tahija project. The pyriproxifen use dropped significantly in the control group (Table 5, action item 4) because people got tired of putting chemicals into their bathing water; in contrast, in the forum meetings of the intervention communities the nature of pyriproxifen was explained and discussed.

**Table 6:** “What kind of community actions did you participate in since March 2009?” (without mentioning any options)

Community actions	Before		After		Difference of difference
	Control N = 210	Intervention N = 213	Control N = 200	Intervention N = 201	
Participation in collective community actions to prevent and control dengue	15.50%	22.90%	57%	88.60%	24.20%
Cleaning up the environment in the neighborhood	87.10%	62.70%	88.60%	89.90%	25.70%
Joining meetings to discuss dengue	9.70%	45.10%	22.80%	74.70%	16.50%
Checking water containers in houses/public spaces to prevent insects from breeding	2.00%	3.00%	1.35%	16.30%	13.95%
Managing household solid waste	0%	0%	0%	9.60%	9.60%
Educating others	0%	2%	1.80%	9%	5.20%
Others	0%	0%	0.90%	24.70%	23.80%

#### Results on vector densities

The study was not designed for determining the efficacy of the intervention on vector abundance, however, the following findings are worth mentioning: The original larval and pupal indices before the Tahija activities and this intervention project showed high values: the Breteau index (number of infested containers in 100 houses) was 29.1 and the pupae per person index (PPI, number of Aedes pupae encountered in a neighborhood per person living there) was 0.37. The pyriproxifen treatment applied by the Tahija foundation in our pre-intervention phase achieved already a substantial reduction of the entomological indices, but there was no control group (the Breteau index declined from 29.1 at baseline to 4.4 after pyriproxifen treatment and the PPI declined from 0.37 to 0.03). There seemed to be an added value of our community-centred project: While no statistical significance testing could be done due to the low number of clusters, calculation of the difference of differences showed the pupae per person index declined from 0.015 to 0.005 in the intervention group, and from 0.05 to 0.04 in the control group. The Breteau index declined from 6.9 to 0.5 in the intervention group and increased from 2.0 to 3.5 in the control group.

## Discussion

The intervention described here included a range of activities that were mainly based on facilitating community forums and working with a number of stakeholders. The activities required only minor financial contributions during the “warming up” phase (for supporting assemblies and volunteers), which could easily be covered by the public sector. There source mobilization was later taken over by the communities and their representatives during the maintenance stage. Our “action research” resulted in an increased involvement of communities in vector control operations as measured not only through interview surveys but also in a descriptive way according to the suggestions of Draper *et al.*(2010).

Outcomes of the intervention included: increased community knowledge, attitude and practices in dengue prevention; increased household and community participation; prospects for sustainability and continued partnership; routine community vector control efforts refocused on environmental and health issues (e.g. cleaning bath water containers, sorting solid waste and making compost, maintaining water and environmental conditions, using repellent plants, cleaning up stagnant water); community ownership; and broader community development activities including solid waste management and recycling.

The strength and weaknesses of the community-centred approach used here, with broad stakeholder involvement, are presented in Table 7.

**Table 7:** Internal and external prospects and limitations of the horizontal approach (community and partnership model) in dengue vector management

Strengths and weaknesses (internal)	1. Sustainability is more promising when people themselves contribute either financially or with in-kind support
	2. Requires greater investment at the beginning (for capacity building at grassroots level) but is cheaper in the later stages when the community voluntarily shares resources
	3. Requires strong leadership, particularly at the beginning, and the ability to liaise among stakeholders including the community
	4. Coverage is limited at the beginning so the approach is less useful for short-term interventions
	5. Slower than vertical approaches to achieve the expected outcome
Opportunities and threats (external)	1. Slow in the beginning to achieve inter-sectoral support, but easier once the inter-sectoral communication channel is stronger
	2. Opportunity to expand to other issues beyond the project focus
	3. Relies on local leadership and community organization

It can be seen that the community-centred multi-stakeholder approach needs more efforts at the beginning but has better prospects for sustainability than the vertical “top–down” approach, which achieves high coverage levels at the start but needs a maintained effort on the side of the public control services. Further research (particularly on cost-effectiveness and long-term sustainability) and practical applications in other settings are needed to gain multi-site experiences with the approach described here.
